# In-Silico UHPLC Method Optimization for Aglycones in the Herbal Laxatives *Aloe barbadensis* Mill., *Cassia angustifolia* Vahl Pods, *Rhamnus frangula* L. Bark, *Rhamnus purshianus* DC. Bark, and *Rheum palmatum* L. Roots

**DOI:** 10.3390/molecules22111838

**Published:** 2017-10-27

**Authors:** Nadja Meier, Beat Meier, Samuel Peter, Evelyn Wolfram

**Affiliations:** Phytopharmacy & Natural Products Research Group, Institute of Chemistry and Biotechnology, Zuerich University of Applied Sciences (ZHAW), CH-8820 Waedenswil, Switzerland; nadja.meier@zhaw.ch (N.M.); xmbe@zhaw.ch (B.M.); Samuel.peter@zhaw.ch (S.P.)

**Keywords:** anthraquinone aglycones, aloe-emodin, rhein, emodin, chrysophanol, physcion, herbal laxatives, ultra high performance liquid chromatography (UHPLC), design of experiment (DOE), chromatographic modelling

## Abstract

For the European Pharmacopoeia (Ph. Eur.) herbal monograph draft of *Cassia angustifolia* Vahl. and *Cassia senna* L. leaves and pods, a safety limitation of aloe-emodin and rhein was proposed, due to toxicological concerns. A quantitative, analytical method of the anthraquinone aglycones in all Ph. Eur. monographed herbal laxatives is of interest. A rational method development for the aglycones aloe-emodin, rhein, emodin, chrysophanol, and physcion in five herbal drugs was realized by using 3D chromatographic modelling (temperature, solvent, and gradient time) and design of experiment (DOE) software (DryLab^®^ 4). A methodical approach suitable for the challenging peak tracking in the chromatograms of the herbal drugs in dependence on the changes in the chromatographic conditions is described by using a combination of mass spectroscopy (MS) data (UHPLC-QDa), UV/Vis-spectra, and peak areas. The model results indicate a low robust range and showed that with the selected chromatographic system, small interferences could not be averted. The separation achieved shows a pure UV/Vis spectrum for all aglycones except for chrysophanol in *Aloe barbadensis* and emodin in *Cassia angustifolia* fruit. A gradient with the best resolution of the aglycones in all five drugs is proposed, and its suitability demonstrated for the quantification of aglycones in these herbal drugs.

## 1. Introduction

**Figure 1:**
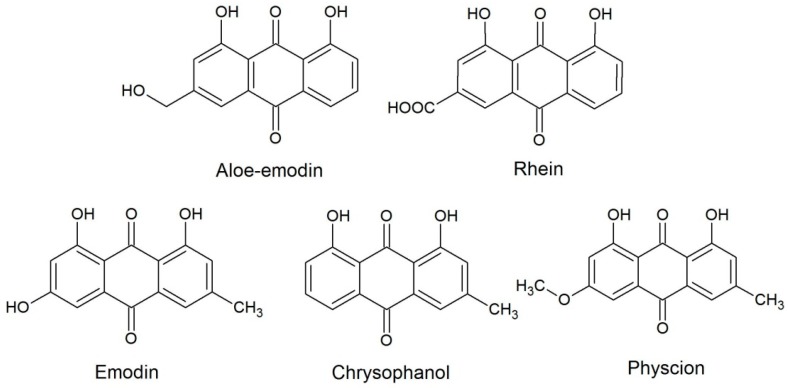
Chemical structure of the anthraquinone aglycones: aloe-emodin, rhein, emodin, chrysophanol, and physcion.

The concentrated and dried juice of the leaves of *Aloe barbadensis* Mill., the pods of *Cassia angustifolia* Vahl., the bark of *Rhamnus frangula* L. and *Rhamnus purshianus* DC., and the roots of *Rheum palmatum* L. belong to the group anthraquinone containing drugs with laxative properties. All are listed in the European Pharmacopoeia (Ph. Eur.). Their laxative effect is based on the anthraquinone glycosides or dianthron glycosides. The metabolism is not well studied but the anthraquinone glycosides or dianthron glycosides are split by colonic bacteria into the free and biologically active aglycones [1]. Depending on the plant species, these anthraquinone aglycones are aloe-emodin, rhein, emodin, chrysophanol, and/or physcion (Figure 1). On the one hand, these aglycones exert numerous biological effects and potentially other effects relevant to pharmacology, for example, anticancer, anti-inflammatory, and antifungal effects [2,3,4,5]. On the other hand, aloe-emodin and emodin show some genotoxic effects in vitro [6,7,8,9] which were not visible in vivo (aloe-emodin) [6,10,11] or herbal extracts [12,13,14,15]. However, a limitation of aloe-emodin and rhein was proposed in the herbal monograph draft for *Cassia angustifolia* Vahl. and *Cassia senna* L. leaves and pods [16]. In light of a potential limitation in *Cassia*, the aglycone content used in other herbal laxatives would be of interest, which leads to the demand for a quantitative analytical method to review the aglycones in all considered herbal laxatives.

For the five aglycones, aloe-emodin, rhein, emodin, chrysophanol and/or physcion, numerous high performance liquid chromatography (HPLC) methods have been published for different species, i.e., *Cassia* sp. [17,18] (HPLC-ultraviolet detection), *Rheum* sp. [19,20,21,22] (HPLC photodiode array detector, HPLC-DAD), *Polygoni* sp. [23] (HPLC-fluorescence detection), and *Rhamnus* sp. [24,25,26] (HPLC-DAD). They all differ markedly in the sample preparation, calibration range, mobile and stationary phases, and are, therefore, not a basis for a reliable comparison concerning the distribution and content of the aglycones in the different herbal drugs of interest. Instead of testing all mentioned methods for all the five herbal drugs, a new systematic development approach was chosen. In this report, the development of an UHPLC-DAD method is presented by using 3D chromatographic modelling (temperature, solvent, and gradient time) and design of experiment (DOE) software (DryLab^®^ 4) with 12 variated measuring points. The model calculates multi-dimensional resolutions through which the conditions for a baseline separation can be elucidated. The interferences for the peaks of interest are taken into account by using mass spectroscopy (MS) data for peak tracking in the 12 different input chromatograms. This approach for peak tracking, in combination with chromatographic modeling software, leads to the possibility of developing a fast separation in highly complex samples with unknown substances within the set boundaries (pH, stationary phase, solvents). To the best of our knowledge, no method development for these anthraquinone aglycones in all five herbal drugs has been performed with DryLab^®^. For the anthraquinone aglycones in rhubarb, a method development with DryLab^®^ scouting runs was presented by Liu et al. [22], however, the methodological approach presented in this report differs markedly.

## 2. Results and Discussion

**Figure 2:**
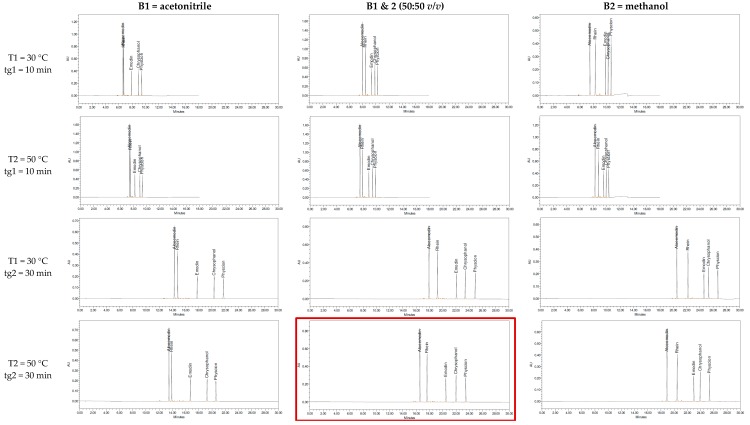
Chromatograms of reference substances aloe-emodin, rhein, emodin, chrysophanol, and physcion for the 12 input runs at 435 nm. tg1: time of gradient 1 (10 min from 1 to 99% B), tg2: time of gradient 2 (30 min from 1 to 99% B)

The most important step for a successful model is the peak tracking of each peak in all 12 input runs. In the case of the reference substances, this is easily achieved. The substances of interest can be injected in pure form, or a mixture with different concentrations of each substance can be used. The second method leads to the risk of difficulties in peak tracking when multiple substances co-elute. However, the aglycones can be easily distinguished due to their mass in combination with the used concentration. The aglycone pairs aloe-emodin and emodin, as well as rhein and physcion, share the same molecular mass. Therefore, higher concentrations were used for aloe-emodin and rhein. A baseline separation was given for the aglycones in ten input runs (Figure 2). Only aloe-emodin and rhein co-eluted with the short gradient (tg1) and acetonitrile as the organic modifier. With the use of methanol, the retention time of all aglycones increased for roughly 2–3 min and the critical peak pair, meaning the pair with the least resolution, shifted from aloe-emodin and rhein to emodin and chrysophanol. The higher temperature increased the elution for roughly 1 min. The aglycones were separated in most conditions and the elution order remained the same in all conditions. However, based on visual examination, the best and most even separation was achieved with the condition tg2 and the mixture of acetonitrile (ACN)/MeOH as an organic modifier (highlighted in Figure 2). Therefore, this condition was chosen as a starting point to determine the interfering peaks from the herbal drugs.

**Figure 3:**
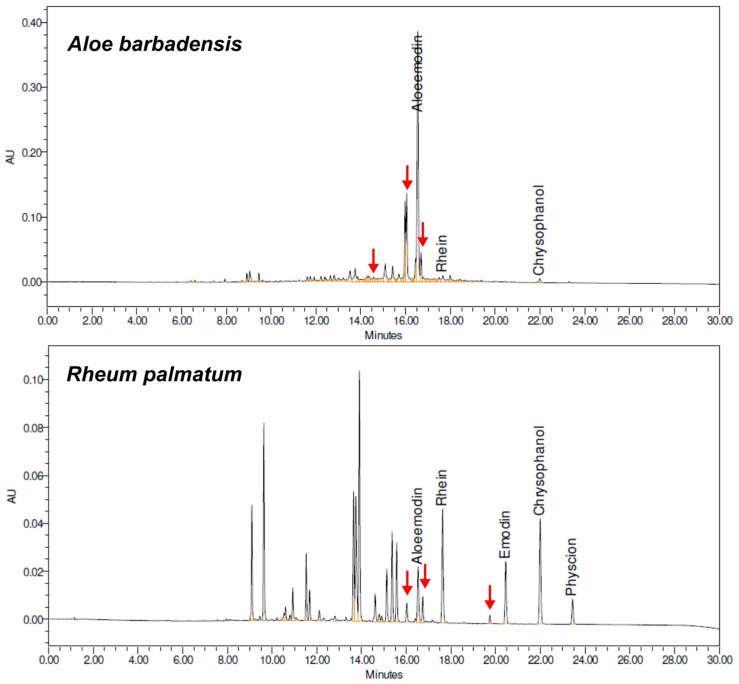
Chromatograms of *Aloe barbadensis* Mill. and *Rheum palmatum* L. roots at 435 nm with conditions T2 (50 °C), tg2 of 30 min, and organic modifier acetonitrile (ACN)/MeOH 50:50 (*v*/*v*). Arrows indicate the peaks which were added to the DryLab^®^ model after successful tracking in all input runs. The chromatograms of these two species were chosen as an example to illustrate the peaks added to the model from the herbal matrix. In the other species, fewer peaks close to the aglycones were added to the model.

In the chromatograms of the herbal drugs, the purity of the aglycone peaks were verified by UV/Vis spectra at multiple points within the peak. If all peaks were pure, the peaks close to the aglycones were chosen and the *m*/*z*-ratio for the base peak in the MS-trace (either positive or negative scan) was determined. Thereafter, the chromatograms at that specific *m*/*z* ratio were derived for the further eleven input runs in order to determine the retention time of the desired peak. When the peak in the other input runs was pure, the identity was checked for plausibility by comparing the UV/Vis-spectra. This procedure was repeated for all peaks of interest from the plant samples. In Figure 3, the chromatograms derived from 435 nm of *Aloe barbadensis* Mill. and *Rheum palmatum* L. roots are shown in the chosen starting point (T2, tg2, mixture of B1 and 2). The retention times of the six peaks, indicated with an arrow, were derived from all input runs by this method. Peaks around the aglycones, as well as peaks eluting at the beginning of the chromatogram, were chosen for the model to create a prediction for the whole herbal matrices. After completing the peak tracking process by this method, 13 additional peaks from the herbal matrices were implemented in the model; three substances from *Aloe barbadensis* Mill., three from *Rheum palmatum* L., three from *Rhamnus frangula* L., one from *Rhamnus purshianus* DC., and three substances from *Cassia angustifolia* Vahl. The retention times of many peaks in the 12 runs were tracked and identified by using this approach. However, there are limitations. Substances which are not ionisable, have no characteristic *m*/*z*-profile or unique mass, as well as peaks which are unseparated in all the input runs, cannot be tracked unless the peak area allows a clear identification in all input runs. Also, for small peak areas, difficulties occur with the peak verification by UV/Vis-spectra.

**Figure 4:**
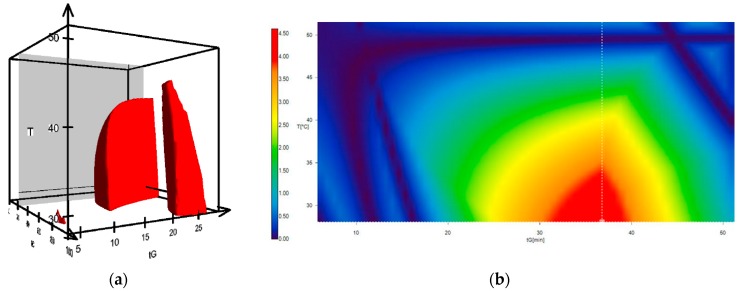
(**a**) Calculated resolution cube showing the working points with a resolution ≥1.5 for the critical peak pair of the 18 peaks subjected to the model. (**b**) Graphical view of the peak resolution in a cross section of the organic eluent acetonitrile/methanol (78:22 *v*/*v*) as a function of the temperature and gradient time. Color scale shows the predicted resolution of the critical peak pair.

Moreover, not all peaks need peak tracking for a successful model prediction. With the 13 peaks from the herbal matrices and the five peaks from the aglycones, a resolution cube with one million chromatograms (100 at each axis of the cube) for a one-step gradient was calculated. The working areas with a resolution ≥1.5 for the critical peak pairs (pair with the lowest resolution per condition) are shown in Figure 4, left. The model output elucidates a very narrow robust range for the separation of the aglycones in all five drugs. The working space with the highest robustness was predicted to be with 22% MeOH/78% ACN as an organic modifier, a temperature of between 30 and 35 °C, and a linear gradient of around 35 min (red area in Figure 4, right). From this linear gradient at a temperature of 30 °C, five gradients were manually optimized within the software to obtain a faster and more precise separation in the aglycones area. The predicted retention times and chromatograms were in good correlation with the ones measured. Additionally, the suitability of the model was shown with sennosides and aglycones in a previous work with *Cassia* under very similar chromatographic conditions [27].

**Figure 5:**
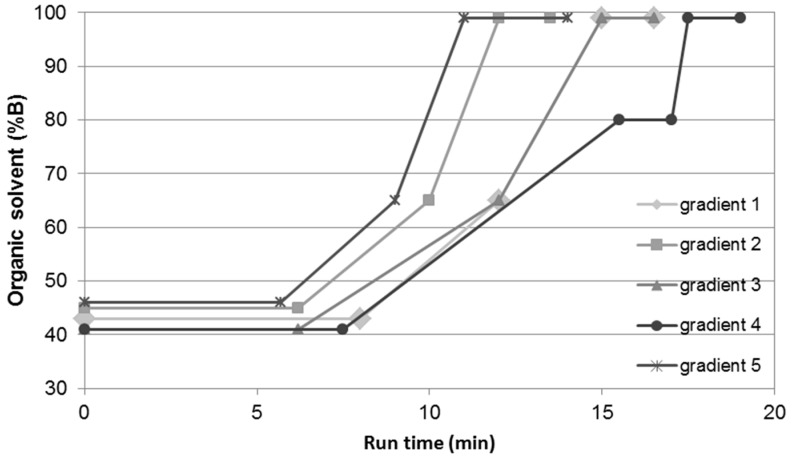
Selected gradients at working point 22% MeOH in ACN as an organic modifier and column temperature of 30 °C.

The five gradients chosen had a run-time of between 13.5 and 19 min (Figure 5) with a predicted minimal resolution >1.8 for the critical peak pair. For the critical peak pair, substances other than the aglycones were selected to obtain a maximal resolution in the area of the aglycones. The selected gradients differed in the starting concentration of the organic modifier (35 to 46% B), the onset time, and gradient slope, as well as in the number of gradient steps. For the test runs, all herbal samples were freshly prepared with a tenfold increase in ratio of drug to extraction solvent (DSR of 100 mg per 1 mL) due to the low content of aglycones in the drugs which resulted in small peaks.

**Figure 6:**
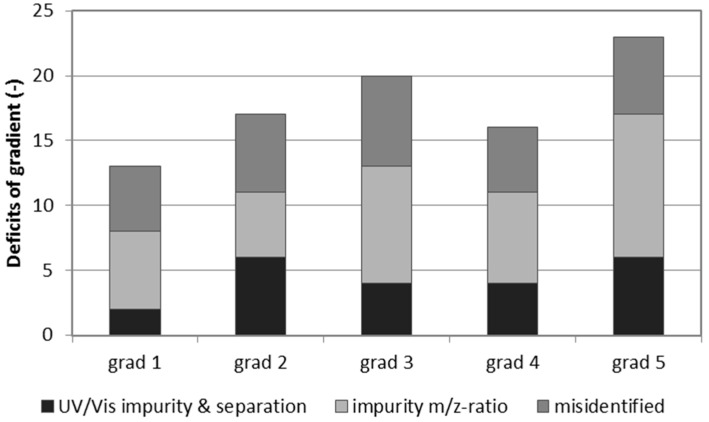
Sum of aglycone peak impurities and misidentified peaks per gradient tested. Each impurity or misidentified aglycone peak was counted as one deficit of the gradient tested.

The obtained data from the test runs was evaluated in three steps. The first evaluation was for peak separation and purity of the peaks. Therefore, the UV/Vis spectra of the aglycones in the herbal samples were analyzed by screening through each peak and studying the peak spectra at the apex, inflections, and valleys. The resulting amount of impure, or not well separated, peaks was counted for each method (Figure 6). As a second approach, the purity of the *m*/*z*-ratio was studied for each aglycone peak as the amount of *m*/*z*—impurities could differ compared to the ones from the UV/Vis spectra due to the underground matrix present in some samples. Lastly, the number of misidentified peaks from the processing step with the software was studied for each herbal sample in order to test the separation for multi-herbal analysis. The sum of those parameters is shown in Figure 6. No gradient tested showed a perfect resolution for all aglycones in all samples.

**Figure 7:**
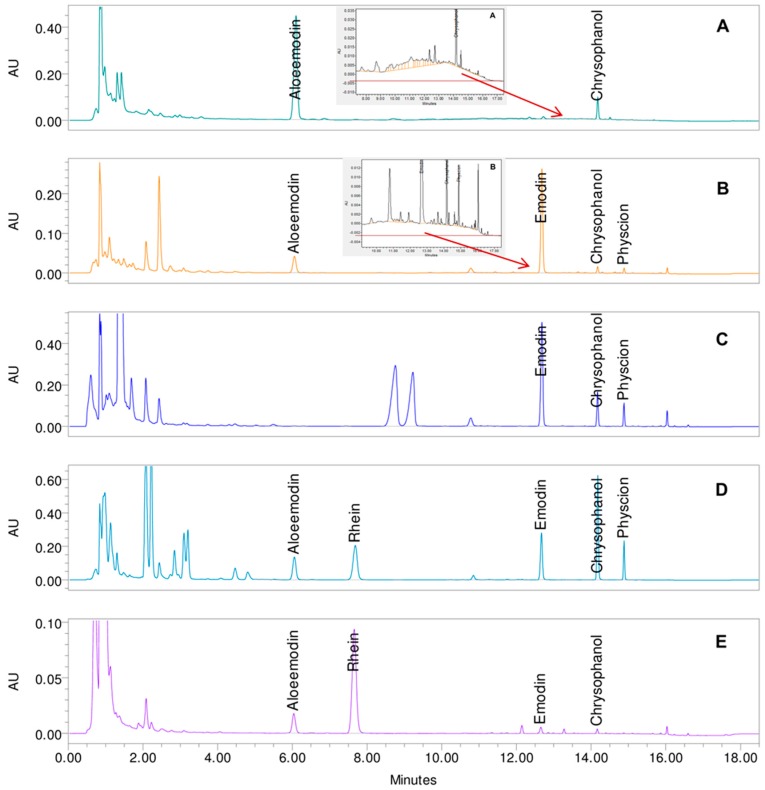
Chromatograms at 435 nm with the selected gradient 1 of the five drug samples (drug solvent ratio 100 mg:1 mL) (**A**) dried leaf juice of *Aloe barbadensis* Mill. (with detailed view: 7.5–17 min); (**B**) bark of *Rhamnus purshianus* DC. (with detailed view: 9–17 min); (**C**) bark of *Rhamnus frangula* L.; (**D**) roots of *Rheum palmatum* L.; (**E**) pods of *Cassia angustifolia*.

The ideal method was considered to have no impurities in the aglycone peaks and no wrongly integrated peaks, meaning no co-eluting substances in all tested herbals allowing for a multi-drug analysis. No gradient tested fulfilled these demands. An addition of further interfering peaks to the model would yield no additional optimizations for the separation due to the already very small robust range of the model (Figure 7). Therefore, gradient 1 was chosen as the best fitting method as this gradient showed the least amount of impurities. The resolutions of the aglycones for the five investigated gradients are shown in Table 1 for the reference solution mix and the five herbal samples investigated. The resolutions are not shown for all peak pairs. Small peaks (height down to 0.0003 AU) were considered that showed an inadequate peak shape, meaning the inflections were not detectable, or no baseline separation. Therefore, the width at a peak height of 50% was not derivable for all peaks, which made the calculations impossible for some peak pairs (indicated as n/a). The height chosen for the integration correlates with the limit of detection based on signal-to-noise ratio the (S/N) of gradient 3. The peak pairs investigated showed strong differences in peak height and also varied in their symmetry. Therefore, the resolutions have to be regarded cautiously. Nevertheless, gradient 1 showed, overall, the best resolutions and no impurities for aloe-emodin.

Gradient 1 showed the best results with only two minor impurities in the aglycone area: one due to the underground matrix in *Aloe barbadensis* interfering with chrysophanol and the second an impurity for emodin (shoulder in UV/Vis spectra at 388 nm) in *Cassia angustifolia*. Due to the low content of aglycones, as well as to the underground matrix, additional peaks in the *m*/*z*—ratio were visible, and small peaks are misidentified at the retention time of aglycones in all herbals except *Rhamni purshiani* and *Rheum palmatum*. The misidentified peaks are rather small (2/3 have a peak height <0.001 AU) and are, therefore, considered to be a minor interference of the aglycones (general peak height of 0.1 to 0.6 AU). Hydroxyanthracene glycosides as well as other polar substances are flushed through the column and elute between 1 and 3 min, as can be seen in Figure 7. The elution of the aglycones starts with aloe-emodin at 6 min and ends with physcion at 14.9 min. The elution of emodin was prolonged to allow a separation from rhein of the two peaks in *Rhamnus frangula* L. between rhein and emodin (frangulin A and B). Overall, with gradient 1 (0/43, 8/43; 12/65; 15/99; 16.5/99 (min/%B)) the separation of the aglycones was very good in all samples. The main disadvantage is the impurities due to the background matrix especially in *Aloe barbadensis* (retention time 9 to 16 min) but also slightly in *Rhamni purshiani* which is only removable through changes in sample preparation.

## 3. Materials and Methods

For the development, a method for anthraquinone glycosides and aglycones in the bark of *Rhamnus frangula* served as a basis in terms of sample preparation, choice of pH condition, and considered solvents for the model [28,29].

### 3.1. Chemicals

Aloe-emodin (>96%, Lot. No. 6479), emodin (>92%, Lot. No. 8668), and physcion (>98%, Lot. No. 2910) were purchased from PhytoLab GmbH & Co. KG (Vestenbergsgreuth, Germany). Rhein (>98%, Lot. No. SLBC4285V) and chrysophanol (>99%, Lot. No. BCBL2252V) were obtained from Sigma-Aldrich (Buchs, Switzerland). Acetonitrile and methanol used were of MS grade (Merck, Darmstadt, Germany) as was formic acid (Carlo Erba Reagents S.r.l., Cornaredo, MI, Italy). Dimethyl sulfoxide (DMSO) was of pure quality (>99%, Sigma-Aldrich, Buchs, Switzerland). Water was of purified quality (Arium^®^ pro VF, Sartorius Stedim Siotech S.A., Tagelswangen, Switzerland).

### 3.2. Standard Solutions

Emodin was dissolved to 0.15 mg/mL in methanol. Aloe-emodin and rhein were dissolved to 0.3 mg/mL and physcion to 0.15 mg/mL in methanol/dimethyl sulfoxide (9:1 *v*/*v*). A higher amount of DMSO was necessary to obtain a clear solution of 0.15 mg/mL for chrysophanol (methanol/dimethyl sulfoxide (6:4 *v*/*v*)). The solutions were mixed for the injection as follows: 3 parts aloe-emodin, 2 parts rhein, 1 part emodin, 1 part chrysophanol, and 1 part physcion, resulting in a final concentration of 112.5 mg/L for aloe-emodin, 75 mg/L for rhein, and 18.75 mg/L for emodin, physcion, and chrysophanol.

### 3.3. Plant Material and Sample Preparation

*Aloe barbadensis* (Lot. No. 0032/1-5-2 F2), *Rhamni purshiani* bark (Lot. No. 23391), and *Rheum palmatum* roots (Lot. No. 23391) were provided by Alfred Galke GmbH (Bad Grund, Germany). *Rhamnus frangula* bark (Lot. No. 136255) and *Cassia angustifolia* pods (Lot. No. 131352) were provided by DIXA AG (St. Gallen, Switzerland). One hundred milligrams respectively 1.0 g of the ground plant material were dispersed in 10 mL acetonitrile/0.2% sodium hydrogen carbonate (68:32 *v*/*v*) in volumetric glass flasks (25 mL) and sonicated for 20 min at 35–40 °C. After cooling down to room temperature, the extract was adjusted to the mark with acidified water (pH 2 by *o*-phosphoric acid, 85%) and filtered over Chromafil^®^ PET 20/25 (0.2 µm pore size, Machery-Nagel AG, Oensingen, Switzerland) into brown glass vials.

### 3.4. Equipment and Fixed Chromatographic Conditions

An Acquity classic UHPLC system (VDwell: 0.145 mL) with Empower 3 software was used for the separations (Waters AG, Baden, Switzerland) consisting of the following modules: Sample Manager, Column Manager, Binary Solvent Manager, PDA eλ Detector (200–500 nm, bandwidth 1.2 nm), Isocratic Solvent Manager and QDa Detector (ESI±, cone: 15 V, capillary: 0. 8kV, 150 to 850 *m*/*z*). The development was performed on ACQUITY UPLC HSS T3 column (1.8 µm, 2.1 × 100 mm, S/N 01463312815724) with a flow rate of 0.3 mL/min. After the column, a splitter was installed in the Isocratic Solvent Manager which transports 9/10 of the eluate to the PDA detector and 1/10 to the QDa Detector mixed with 0.2 mL/min makeup solvent of acetonitrile/water/formic acid (50:50:0.05 *v*/*v*/*v*). The injection volume was 5 µL.

### 3.5. Model Parameters

The DryLab^®^ 4 software (version 4.2.0.3, Molnár-Institute, Berlin, Germany) uses algorithms based on thermodynamic models of the chromatographic process and is well-established for the optimization of complex chromatographic procedures. The commercially available software is suitable for the Quality by Design (QbD) approach and validations of the model have been carried out by the Molnár Institute (distributor of the model) and through its use world-wide [30,31,32].

**Figure 8:**
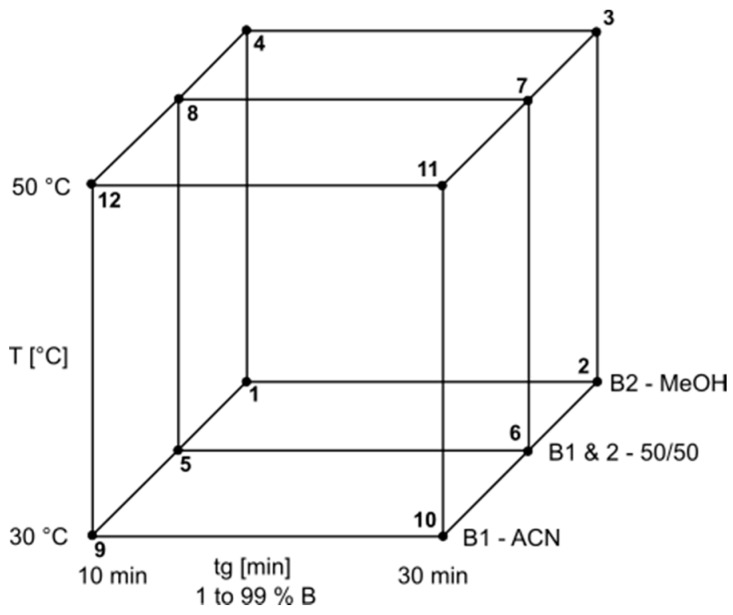
Design of experiment for the model of temperature (T), gradient time (tg), and organic modifier (B1–2). ACN: acetonitrile; MeOH: methanol; B1 and 2: mixture of both 50:50 *v*/*v*, all solvents with addition of 0.1% formic acid.

The model needs retention data from so called “input-runs”. Depending on the model, two, four or twelve runs under certain conditions have to be collected prior to using the software. The ternary model was chosen for the development, which means the parameters gradient slope (tg), column temperature (T), and composition of the organic modifier (B) were varied for the input runs. Formic acid (0.1% *v*/*v*) was added to all mobile phases which allowed for a model independent from pH changes due to the gradient. Also, a lower pH is beneficial for the separation and peak form due to ionization suppression of the carboxyl group of rhein. The gradients were run from 1% organic modifier (B) to 99%. Water with 0.1% formic acid was used as a weak solvent (A). Two gradient times (tg) were applied (tg1 with 10 min and tg2 with 30 min), as well as two input temperatures (T1 with 30 °C and T2 with 50 °C). The design of the input parameters resulting in the twelve runs can be seen in Figure 8.

## 4. Conclusions

The methodical approach of combining the chromatographic modelling and DOE software (DryLab^®^ 4) with UHPLC-QDa proved to be very helpful in developing a gradient method for aglycones in five herbal drugs in as short a time as possible. The peak tracking for the unknown herbal substances worked for almost all peaks of interest and were relevant for the model by using the chromatogram with the best resolution as a starting point and the *m*/*z*-ratio in the other input runs. The changes of the retention time of each peak within the 12 input runs are also very useful for a general verification of a substance’s presence in a complex herbal mixture, i.e. small peak areas of the substances lead to unreliable UV/Vis-spectra. The presented approach is a fast way of showing the limits of a selected chromatographic system in the optimization process, which is very useful for rational UHPLC development in complex herbal mixtures. The robust range of the presented model showed that a perfect method is not possible by using the selected chromatographic system, as small interferences could not be averted. Nevertheless, the separation achieved shows a pure UV/Vis spectrum for all aglycones except for chrysophanol in *Aloe barbadensis* and emodin in *Cassia angustifolia* fructus. As the interferences are very low in their signal intensity compared to the major aglycone peaks, the method is still suitable for the quantification of aglycones in herbal mixtures with adequate errors computation. The risk of other interfering substances is still a possibility as only one herbal sample per herbal drug was used for the study. The approach presented could be optimized by using a blend of different batches per herbal drug and/or a blend of all herbs of interest as, for example, batches from different geographic origins or different plant parts. The method presented still needs validation in terms of robustness, linearity, accuracy, and precision which will be presented in another report.

## Figures and Tables

**Table 1:** Resolution of the aglycones in each sample for the five investigated gradients. The resolution was calculated manually at 50% width and small peaks (>0.0003 AU) were considered for the calculation of the resolution.

		Gradient 1	Gradient 2	Gradient 3	Gradient 4	Gradient 5
**Reference solutions**	**AE**					
	**R**	7.19	6.48	7.88	8.73	6.06
	**E**	29.33	27.79	28.22	25.66	27.23
	**C**	17.66	17.18	18.85	18.31	16.39
	**P**	11.97	9.97	11.47	12.85	9.70
***A. barbadensis***	**AE**	2.22	impure	n/a	n/a	1.74
	**C**	n/a	impure	impure	2.22	n/a
***R. purshianus***	**AE**	1.44	n/a	2.36	2.15	1.73
	**E**	4.51	4.17	5.25	4.91	n/a
	**C**	1.97	impure	2.19	3.07	impure
	**P**	1.65	n/a	1.70	1.20	n/a
***R. frangula***	**E**	n/a	n/a	n/a	n/a	5.52
	**C**	4.76	n/a	4.97	5.05	n/a
	**P**	2.49	n/a	impure	n/a	n/a
***R. palmatum***	**AE**	2.68	2.75	2.65	impure	impure
	**R**	5.31	4.84	n/a	n/a	4.12
	**E**	4.13	4.65	5.59	n/a	impure
	**C**	3.26	3.52	3.17	13.08	2.68
	**P**	impure	impure	impure	impure	impure
***C. angustifolia***	**AE**	2.77	1.41	n/a	n/a	2.76
	**R**	5.65	5.12	n/a	impure	5.28
	**E**	2.10	impure	1.70	1.88	impure
	**C**	impure	2.27	impure	impure	1.73

AE: aloe-emodin, R: rhein, E: emodin, C: chrysophanol, P: physcion, n/a: peak height too small and/or separation insufficient to derive width, impure: co-elution, not calculated.
